# Supplementary material for: Mycophenolate mofetil reduces the branching of microglial processes
Source: Mol Brain. 2026 Jan 11;19:12. doi: 10.1186/s13041-025-01271-1 (PMC12882203; doi:10.1186/s13041-025-01271-1)
Supplement: Supplementary file 2 — Additional file2. [file 13041_2025_1271_MOESM2_ESM.pdf]

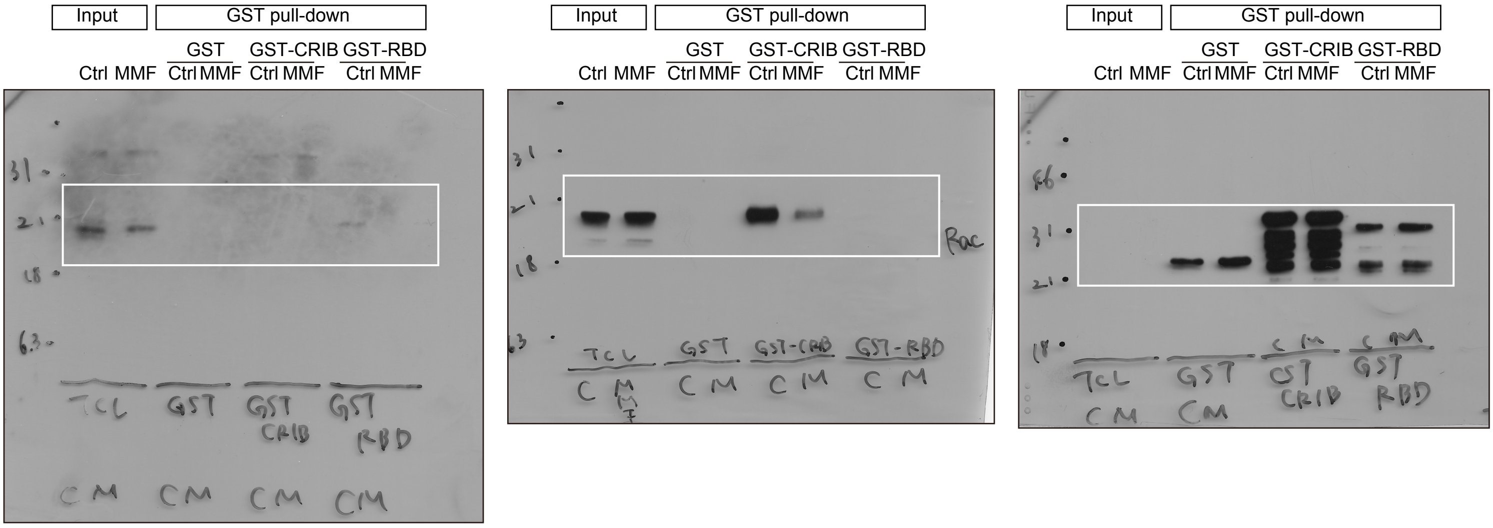

Supplementary Data 1
Overall images of the gel in the Fig.3B before cropping

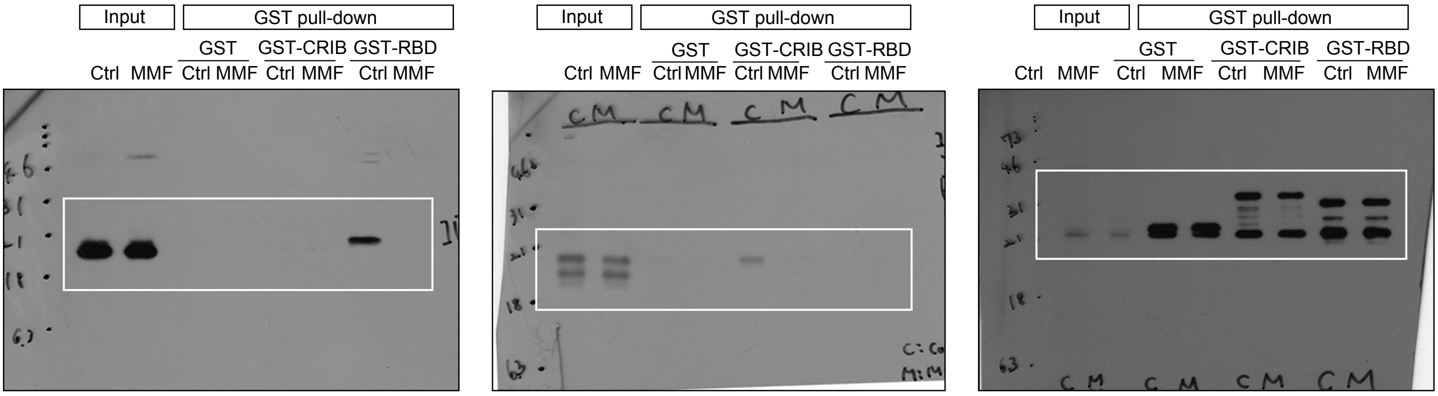

Supplementary Data 2
Overall images of the gel in the Fig.4A before cropping

Parameters	
Polarity	Negative
Pitch	50 μm
m/z range	m/z 350-550
DL temperature	290°C
Heat block temparature	450°C
Sample voltage	-4.50 [kV]
Detector voltage	2.16 + 0.2 [kV]
Repetition number	1000 [Hz]
Laser shots	50 [shots]
Laser diameter	4
Laser power	68.0

Supplementary Table 1
Parameters used for the optimization and data acquisition
